# LRP5-/6 gene polymorphisms and its association with risk of abnormal bone mass in postmenopausal women

**DOI:** 10.1186/s13018-023-03829-y

**Published:** 2023-05-18

**Authors:** Jun Li, Zebing Liu, Yanxia Ren, Han Shao, Siyuan Li

**Affiliations:** 1 The First Affiliated Hospital of Shihezi University, 107 North Second Road, Hongshan Sub-District, Shihezi City, 832000 Xinjiang Uygur Autonomous Region People’s Republic of China; 2 School of Medicine, Shihezi University, Shihezi, 832000 Xinjiang Uygur Autonomous Region People’s Republic of China

**Keywords:** LRP5-/6, Abnormal bone mass, Gene interaction, Single-nucleotide polymorphism, Haplotype

## Abstract

**Objectives:**

To assess LRP5-/6 gene polymorphisms and its association with risk of abnormal bone mass (ABM) in postmenopausal women.

**Methods:**

The study recruited 166 patients with ABM (case group) and 106 patients with normal bone mass (control group) based on bone mineral density (BMD) results. Multi-factor dimensionality reduction (MDR) was used to analyze the interaction between the Low-density lipoprotein receptor-related protein 5 (LRP5) gene (rs41494349, rs2306862) and the Low-density lipoprotein receptor-related protein 6 (LRP6) gene (rs10743980, rs2302685) and the subjects’ clinical characteristics of age and menopausal years.

**Results:**

(1) Logistic regression analysis showed that the subjects with the CT or TT genotype at rs2306862 had a higher risk of ABM than those with the CC genotype (OR = 2.353, 95%CI = 1.039–6.186; OR = 2.434, 95%CI = 1.071, 5.531; *P* < 0.05). The subjects with the TC genotype at rs2302685 had a higher risk of ABM than those with the TT genotype (OR = 2.951, 95%CI = 1.030–8.457, *P* < 0.05). (2) When taking the three Single-nucleotide polymorphisms (SNPs) together, the accuracy was the highest with the cross-validation consistency of 10/10 (OR = 1.504, 95%CI:1.092–2.073, *P* < 0.05), indicating that the LRP5 rs41494349 and LRP6 rs10743980, rs2302685 were interactively associated with the risk of ABM. (3) Linkage disequilibrium (LD) results revealed that the LRP5 (rs41494349,rs2306862) were in strong LD (*D*′ > 0.9, *r*^2^ > 0.3). AC and AT haplotypes were significantly more frequently distributed in the ABM group than in the control group, indicating that subjects carrying the AC and AT haplotypes were associated with an increased risk of ABM (*P* < 0.01). (4) MDR showed that rs41494349 & rs2302685 & rs10743980 & age were the best model for ABM prediction. The risk of ABM in “high-risk combination” was 1.00 times that of “low-risk combination”(OR = 1.005, 95%CI: 1.002–1.008, *P* < 0.05). (5) MDR showed that there was no significant association between any of the SNPs and menopausal years and ABM susceptibility.

**Conclusion:**

These findings indicate that LRP5-rs2306862 and LRP6-rs2302685 polymorphisms and gene–gene and gene–age interactions may increase the risk of ABM in postmenopausal women. There was no significant association between any of the SNPs and menopausal years and ABM susceptibility.

## Introduction

Osteoporosis (OP) is a metabolic disease, which is characterized by decreased bone density and damaged bone microstructure [1]. Postmenopausal women comprise about 1/3 of the 8.3 million people in China suffering from OP [2]. OP is a complex systemic disease resulting from genetic and environmental factors interacting. Biochemical markers of bone turnover (BTMs) are commonly used for therapy monitoring purposes for osteoporotic patients [3]. Currently, more than 400 gene loci associated with osteoporosis have been found [4, 5]. Among them, the effects of SNP of LRP5 and LRP6 genes on OP have attracted much attention. A study showed that LRP6 polymorphism may be associated with body composition and BMD in Iranian children [6]. Another study showed that there is a modest effect of the LRP5 rs3736228 C > T on the increased susceptibility of osteoporosis [7]. Moreover, the study [8] found that bone mass and bone strength decreased with age, indicating that age played an important role in BMD. In addition, estrogen is one of the important factors affecting OP, postmenopausal hormonal modification negatively impacts the quality of the bone [9]. Several studies have shown that with the increase in menopausal years, the risk of osteoporosis increases [10–12]. In our previous study, we investigated that the polymorphism of LRP5 gene was related to the decrease in BMD in postmenopausal women [13]. In order to further explore LRP5-/6 gene polymorphisms and its association with risk of abnormal bone mass in postmenopausal women, we performed a case–control study to investigate the role of LRP5-/6 gene–gene, gene-age and gene-menopausal years interactions on the risk of ABM in postmenopausal women.

## Materials and methods

### Study subjects

A total of 166 ABM patients and 106 age-matched healthy controls were included in this study. Patients with ABM were consecutively enrolled from the First Affiliated Hospital of Shihezi University School of Medicine between December 2018 and December 2019. The diagnostic criteria for OP refer to guidelines established by WHO in 1994 [14]. The controls were also registered from the same hospital during the same period. Based on the results of BMD, the study subjects were divided into the control group (normal bone mass group) and the ABM group (T-score ≤ -2.5SD for OP, − 2.5SD < T score < -1.0SD represents reduced bone mass; if T score < -1.0SD, represents abnormal bone mass (ABM). Inclusion criteria. (1) Women who have been in natural menopause for more than one year; (2) Ages 47 to 80 years; (3) All the participants were Han nationality living in Xinjiang Uygur Autonomous Region, without any genetic relationship, and had a complete medical history. Exclusion criteria: autoimmune diseases, thyroid, and parathyroid diseases, severe cardiac, hepatic, and renal diseases, and malignant tumors with underlying diseases affecting Ca and other bone metabolic indexes. This study was performed by the ethical guidelines of the Helsinki Declaration of 1975 (revised in 2008). It was approved by the Ethics Committee of the First Affiliated Hospital of Shihezi University School of Medicine (ethics number:2019–129-01). All participants signed informed consent forms.

### Observation indicators

General descriptive data of the subjects: age, menopausal years, body mass index (BMI), and waist-to-hip ratio (WHR) of the included study subjects were collected and analyzed. The concentrations of peripheral serum calcium (Ca), phosphorus (P), and alkaline phosphatase (ALP) were measured by Roche automatic biochemical analyzer (Model Modular DPPH7600).

BMD of the lumbar spine 1–4 (L1-4) and femoral neck (FN) was measured by dual-energy X-ray (DEXA).

### Genotyping

Genomic DNA was extracted from the peripheral blood of the participants using the DNeasy Blood & Tissue Kit(Thermo Fisher Scientific (China) Equipment Co) and stored at − 80 °C for future experiments. The Agena Mass ARRAY system was used for SNP genotyping. The primers for each locus are shown in Table 1.

**Table 1:** Primers and PCR products

SNPs	Primers	PCR product length/bp
LRP5-rs41494349	CCGCAGTGGACTTCCAGTTT	88
	CGTCTGGTTCAGGTAGGTCG	
LRP5-rs2306862	AGTTTGGCCTTGACTACCCC	95
	GCGCCACTTCGATTCTTTGG	
LRP6-rs10743980	CCCTTATCCGAACTGAAAACACC	71
	GGATTTCTTTCTGCAGGATGGC	
LRP6-rs2302685	TGAGGAGAGTCTCAGAAGCCA	80
	TCGAGCCTTGTGCTAAACCC	

*SNP* single-nucleotide polymorphism

### Statistical analysis

Statistical analyses were performed using the SPSS 22.0 software. K-S method was used to test the normality of all data. The measurement data that do not conform to the normal distribution were expressed by quartiles, and rank sum test was used for comparison between two groups. The *χ*^2^ test was used to determine whether the LRP5 and LRP6 genes conformed to the Hardy–Weinberg equilibrium and the frequency of genotype and gene distribution between groups. Logistic regression analysis was used to analyze the association between gene polymorphism and ABM susceptibility. Multidimensional dimension reduction (MDR) was used to establish models and detect gene–gene, gene-age, and gene-menopausal years interactions. The linkage disequilibrium structure was constructed by SHEsis software, and the genetic association was examined at haplotype level. All tests were bilateral inspections, and a value of *P* < 0.05 was considered statistically significant.

## Results

### Characteristics of the research subjects

This study included 166 ABM patients (case group) and 106 healthy controls (control group). Compared with the control group, the ABM group had higher menopausal years (*P* < 0.05) and lower BMD (L1-4, FN) (*P* < 0.01) (Table 2).

**Table 2:** General characteristics of subjects in ABM and control group

Variables	Control group	ABM group	*P*-value
Age (years)	67.78 (65.97–69.59)	67.88 (65.94–69.82)	0.953
Menopausal years	17.00 (11.50–22.50)	21.00 (18.00–24.00)	0.03*
WHR	0.90 (0.86–0.92)	0.90 (0.85–0.93)	0.22
BMI (kg/m^2^)	26.10 (23.49–28.95)	25.33 (23.00–27.47)	0.70
Ca (mmol/L)	2.26 (2.21–2.32)	2.26 (2.21–2.32)	0.96
P (mmol/L)	1.10 (1.03–1.14)	1.09 (0.99–1.16)	0.87
ALP (U/L)	75.00 (62.00–87.00)	75.00 (61.00–91.00)	0.97
BMD (L_1–4_) (g/m^2^)	1.16 (1.10–1.29)	0.92 (0.82–0.97)	0.00**
BMD (FN) (g/m^2^)	0.89 (0.80–0.94)	0.73 (0.65–0.81)	0.00**

*ABM* Abnormal bone mass, *WHR* Waist-to-hip ratio, *BMI* Body mass index, *ALP* Alkaline phosphatase, *BMD* Bone mineral density

Data were presented as means ± SD for median (interquartile ranges) for continuous variables. Compared with the control group

Italicized value is statistically significant, *P < 0.05, **P < 0.01 indicates statistical significance

### Hardy–Weinberg genetic equilibrium and ABM susceptibility analysis

The Hardy–Weinberg equilibrium (HWE) test was performed in the case group and the control group. All four loci obeyed the HWE(*P* > 0.05).

Logistic regression analysis showed that the subjects with the CT or TT genotype at rs2306862 had a higher risk of ABM than those with the CC genotype (OR = 2.353, 95%CI = 1.039–6.186; OR = 2.434, 95%CI = 1.071, 5.531; *P* < 0.05). The subjects with the TC genotype at rs2302685 had a higher risk of ABM than those with the TT genotype (OR = 2.951, 95%CI = 1.030–8.457, *P* < 0.05) (Table 3).

**Table 3:** Binary logistic regression analysis of LRP5 and LRP6 SNP genotype and ABM susceptibility

Group	Genotype		OR (95% CI)	*P*-value
	ABM	Control		
LRP5-rs41494349				
AA	138(83.13)	92(86.79)	1	1.00
AG	26(15.66)	12(11.32)	1.444(0.512,4.074)	0.487
GG	2(1.20)	2(1.87)	0.667(0.041,10.928)	0.776
AG/GG	28(16.87)	14(13.21)	1.333(0.500,3.556)	0.565
Allele G	30(9.04)	16(7.55)		
LRP5-rs2306862				
CC	108(65.06)	86(81.13)	1	1.00
CT	50(30.12)	16(15.09)	2.353(1.039,6.186)	0.041*
TT	8(4.82)	4(3.77)	2.028 (0.375,10.975)	0.412
CT/TT	28(43.37)	10(18.87)	2.434(1.071,5.531)	0.034*
Allele T	66(24.10)	24(11.32)		
LRP6-rs10743980				
CC	94(56.63)	70(66.04)	1	1.00
CT	64(38.55)	32(30.19)	1.489(0.709,3.130)	0.293
TT	8(4.82)	4(3.77)	1.489(0.258,8.595)	0.656
CT/TT	72(43.37)	36(33.96)	1.489(0.728,3.045)	0.275
Allele T	80(24.10)	40(18.87)		
LRP6-rs2302685				
TT	122(73.49)	90(84.91)	1	1.000
TC	38(22.89)	10(9.43)	2.951(1.030,8.457)	0.044*
CC	6(3.61)	6(3.77)	0.492(0.079,3.068)	0.492
TC/CC	40(26.51)	16(15.09)	2.319(0.911,5.898)	0.078
Allele C	50(15.06)	22(10.38)		

*ABM* Abnormal bone mass, *OR* odds ratio, *95% CI* 95% confidence interval, Data were presented as numbers (proportions)for categorical variables

Adjusted for age and menopausal years

Italicized value is statistically significant, *P < 0.05 indicates statistical significance

### LRP5-/6 gene locus interactions

Table 4 shows interaction analysis among the LRP5 rs41494349, rs2306862 and LRP6 rs10743980, rs2302685. When taking the three SNPs together, the accuracy was the highest with the cross-validation consistency of 10/10 (OR = 1.504, 95%CI:1.092–2.073, *P* < 0.05), indicating that the LRP5 rs41494349 and LRP6 rs10743980, rs2302685 were interactively associated with the risk of ABM.

**Table 4:** MDR analysis of the interaction of LRP5 gene rs41494349, rs2306862 with LRP6 gene rs10743980, rs2302685

Gene–gene interactions		Training balancing accuracy	Testing accuracy	OR (95% CI)	*P*-value	Cross-validation consistency
2	rs41494349&rs2306862	0.556	0.525	0.894 (0.319,2.509)	0.095	8/10
	rs41494349&rs2302685	0.550	0.491	1.571 (0.924,2.672)	0.095	9/10
	rs41494349&rs10743980	0.589	0.486	0.823 (0.405,1.673)	0.671	9/10
	rs2306862&rs2302685	0.535	0.492	2.478 (0.274,22.419)	0.419	9/10
	rs2306862&rs10743980	0.593	0.547	1.462 (1.128,1.895)	0.004**	9/10
	rs2302685&rs10743980	0.601	0.571	0.917 (0.329,2.557)	0.869	10/10
3	rs41494349&rs2302685&rs10743980	0.627	0.475	1.504 (1.092,2.073)	0.013*	10/10
	rs41494349&rs2306862&rs2302685	0.552	0.521	0.458 (0.060,3.513)	0.452	10/10
	rs2306862&rs2302685&rs10743980	0.579	0.567	0.504 (0.800,3.167)	0.465	9/10
4	rs10743980&rs2302685&rs2306862&rs41494349	0.624	0.531	2.763 (0.493,15.506)	0.248	10/10

*SNP* single-nucleotide polymorphism, *OR* odds ratio, *95% CI* 95% confidence interval

Adjusted for age and menopausal years

Italicized value is statistically significant, *P < 0.05, **P < 0.01 indicates statistical significance

### Analysis of linkage disequilibrium

**Fig. 1:**
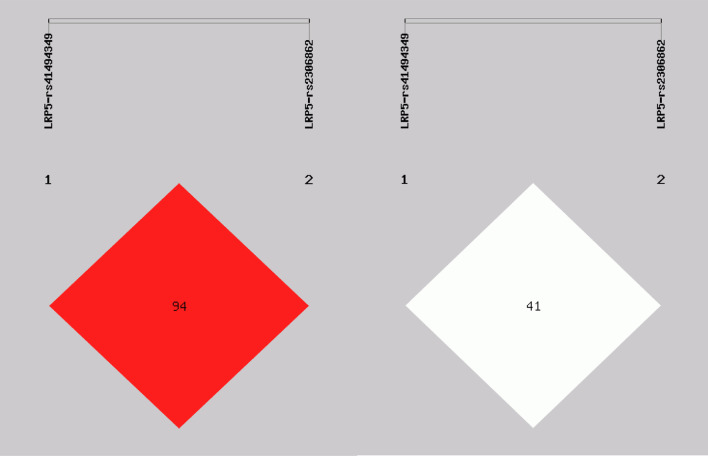
The linkage disequilibrium (LD) among two SNPs in LRP5 gene (*D*ʹ = 0.944, *r*^2^ = 0.415)

Linkage disequilibrium (LD) results revealed that the LRP5 (rs41494349, rs2306862) were in strong LD (*D*′ > 0.9, *r*^2^ > 0.3) (Fig. 1). AC and AT haplotypes were significantly more frequently distributed in the ABM group than in the control group, indicating that subjects carrying the AC and AT haplotypes were associated with an increased risk of ABM (*P* < 0.01) (Table 5).

**Table 5:** Haplotype frequencies of LRP5 gene in ABM patients and healthy controls

Haplotype^#^	ABM (*n* = 166)	Control (*n* = 106)	OR (95%CI)	*P*-value
AC	263.80(0.795)	188.00(0.887)	0.51 (0.308 ~ 0.844)	0.008**
AT	38.20(0.115)	8.00(0.038)	3.34 (1.527 ~ 7.308)	0.001**
GT	27.80(0.084)	16.00(0.075)	1.13 (0.594 ~ 2.140)	0.714
GC	2.20(0.007)	0.00(0.000)		

*ABM* Abnormal bone mass, *OR* odds ratio, *95% CI* 95% confidence interval

^#^The haplotypes of LRP5 gene rs414943349 and rs2306862 loci, *P<0.05, **P < 0.01

(All those frequency < 0.03 will be ignored in analysis)

Italicized value is statistically significant, *P < 0.05, **P < 0.01indicates statistical significance

**Fig. 2:**
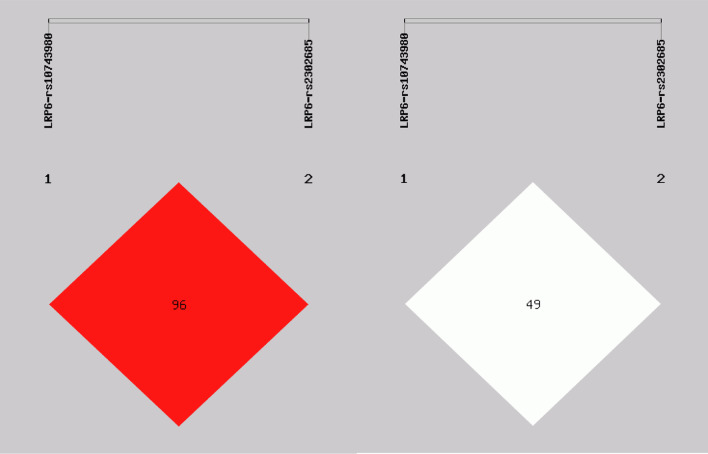
The linkage disequilibrium (LD) among two SNPs in LRP6 gene (*D*ʹ = 0.961, *r*^2^ = 0.498)

Additionally, the LRP6(rs10743980, rs2302685) were in strong LD(*D*′ > 0.9, *r*^2^ > 0.3) (Fig. 2).There was no significant difference in the haplotype between two groups (Table 6).

**Table 6:** Haplotype frequencies of LRP6 gene in ABM patients and healthy controls

Haplotype^#^	ABM (*n* = 166)	Control (*n* = 106)	OR (95%CI)	*P*-value
CC	0.00(0.000)	2.13(0.010)		
CT	252.00(0.759)	169.87(0.801)	0.742 (0.484 ~ 1.136)	0.169
TC	50.00(0.151)	19.87(0.094)	1.695 (0.977 ~ 2.942)	0.058
TT	30.00(0.090)	20.13(0.095)	0.947 (0.518 ~ 1.695)	0.829

*ABM* Abnormal bone mass, *OR* odds ratio, *95% CI* 95% confidence interval

^#^The haplotypes of LRP6 gene rs10743980 and rs2302685 loci, *P<0.05, **P<0.01

(All those frequency < 0.03 will be ignored in analysis)

Italicized value is statistically significant, *P < 0.05, **P<0.01 indicates statistical significance

### Interaction between LRP5-/6 SNPs-age and menopausal years

#### LRP5-/6 SNPs-age interaction

The MDR was used to analyze the interaction between the alleles of LRP5 rs41494349 rs2306862 and LRP6 rs10743980, rs2302685, and the subjects’ age. We observed that rs41494349 & rs2302685 & rs10743980 & age were the best model for ABM prediction. The risk of ABM in “high-risk combination” was 1.00 times that of ‘low-risk combination’ (OR = 1.005, 95%CI: 1.002–1.008, *P* < 0.05) (Table 7).

**Table 7:** MDR analysis of the interaction of LRP5 gene rs41494349, rs2306862 and LRP6 gene rs10743980, rs2302685 alleles with the subjects’ age

Gene-age interaction		Training balancing accuracy	Testing accuracy	OR (95% CI)	*P*-value	Cross-validation consistency
1	rs2306862&Age	0.582	0.541	1.010 (1.003,1.017)	0.004**	9/10
	rs2302685& Age	0.621	0.597	1.016 (1.004,1.028)	0.011*	10/10
2	rs2306862&rs10743980& Age	0.551	0.745	1.008 (0.999,1.016)	0.082	8/10
3	rs41494349&rs2302685&rs10743980& Age	0.574	0.561	1.005 (1.002,1.008)	0.001**	10/10

*SNP* single-nucleotide polymorphism, *OR* odds ratio, *95% CI* 95% confidence interval

Adjusted for menopausal years, *P < 0.05, **P < 0.01

Italicized value is statistically significant, *P < 0.05, **P < 0.01indicates statistical significance

#### LRP5-/6 SNP- menopausal years

The MDR was used to analyze the interaction between the alleles of LRP5 rs41494349 rs2306862 and LRP6 rs10743980, rs2302685 and the subjects’ menopausal years, and we did not observe any significant association between any of the SNPs and menopausal years and ABM susceptibility (*P* > 0.05) (Table 8).

**Table 8:** MDR analysis of the interaction of LRP5 gene rs41494349, rs2306862 and LRP6 gene rs10743980, rs2302685 alleles with the menopausal years

Gene-age interaction		Training balancing accuracy	Testing accuracy	OR (95% CI)	*P*-value	Cross-validation consistency
1	rs2306862&Age	0.514	0.492	1.030 (0.984, 1.078)	0.208	10/10
	rs2302685& Age	0.532	0.517	1.024 (0.994, 1.056)	0.122	9/10
2	rs2306862&rs10743980& Menopausal years	0.578	0.552	1.011 (0.987, 1.035)	0.378	10/10
3	rs41494349&rs2302685&rs10743980& Menopausal years	0.548	0.532	1.013 (0.987, 1.040)	0.324	10/10

*SNP* single-nucleotide polymorphism, *OR* odds ratio, *95% CI* 95% confidence interval

Adjusted for age, *P<0.05, **P<0.01

Italicized value is statistically significant, *P < 0.05, *P<0.01 indicates statistical significance

## Discussion

Postmenopausal osteoporosis is a long-term, progressive disease associated with a high risk of fractures, which seriously threatens people's health [15]. Currently, the most common prescription agent for postmenopausal osteoporosis is Denosuma, which reduces the frequency of non-vertebral fractures and increases bone formation [16, 17].

Wnt signaling pathway is one of the important signaling pathways to regulate bone metabolism [18]. LRP5-/6 receptor protein inhibits bone resorption and promotes bone formation by binding to downstream β-catenin [19], the mutation of LRP5-/6 gene leads to the loss of control of Wnt signal pathway, which affects bone metabolism [20, 21]. OP is not only influenced by genetic factors but also by other factors, such as age and menopause age [22, 23]. However, the pathogenesis of OP cannot be entirely explained by any of the genetic variables that have been found, therefore genetic interaction and gene-age/menopausal years interaction may be another significant cause of the disease.

In the present study, it was found that the risk of ABM in CT and CT/TT genotypes of LRP5 gene at rs2306862 locus was higher than that in CC genotype, indicating that the mutation of allele T increases the risk of ABM. At the rs2302685 locus of LRP6 gene, the risk of ABM in TC genotype was higher than that in TT genotype, indicating that the mutation of allele C increases the risk of ABM. AI et al. [24] found that uncommon loss-of-function mutations facilitate DKK1 competitive binding to the LRP5-/6 receptor in the LRP5 gene, which prevented Wnt/-catenin signaling. Riancho et al. [25] discovered that the polymorphisms at the LRP6 gene locus rs2302685 and rs11054704 were responsible for the decline in BMD in postmenopausal women in Europe. Additionally, Kokubu et al. [26] discovered that people with the CC genotype of the LRP6 gene rs2302685 loci had intercrural BMD that was considerably greater than those with the TT/CT genotype. Animal experiments also revealed that LRP5-/6 are functionally overlapping homologous receptors. In mouse embryonic development, both proteins stimulate postnatal bone acquisition via Wnt signaling stimulates postnatal bone acquisition [27, 28], and LRP6-/- mice were born to die at birth due to mesial skeletal and limb defects, whereas adult LRP5-/- mice result in OP [29]. This conclusion was in line with the present literature reports.

In the present study, we found that the *D*ʹ value between LRP5-rs41494349 and rs2306862 was more than 0.8; it showed a strong chain reaction. Thus, we also conducted haplotype analysis between rs41494349 and rs2306862. The results indicated that the haplotype containing the AC and AT haplotypes was associated with an increased OP risk. Kitjaroentham A et al. [30] found that the risk of osteopenia/osteoporosis was not correlated with either the rs41494349 or the rs2306862 SNPs. This contradicts our findings, and it may be due to the complex interactions between genes, which require further analysis. There was also a strong linkage disequilibrium between the two SNPs at the LRP6- rs10743980 and rs2302685, but haplotype analysis did not show a difference in haplotype frequency distribution between the two groups. This may be because different haplotype interactions can result in different phenotypic effects, which need further analysis.

At the same time, we investigated the interaction of each LRP5-/6 gene locus and found that the polymorphisms of rs2306862&rs10743980, rs41494349&rs2302685 & rs10743980 gene SNP were synergistic with the development of ABM and were risk factors for the development of ABM. Animal studies have shown that mice carrying both LRP5 and LRP6 heterozygous mutations could reduce BMD and cause limb deformities [31]. Van Meurs et al. [32] found that LRP5 and LRP6 genes played a role in bone metabolism and fracture. These findings imply that genetic variations may influence the occurrence of OP. Age and the polymorphisms at the rs2306862, rs2302685, rs41494349, rs2302685, and rs10743980 SNPs were risk factors for the development of ABM. It is generally known that being older is a risk factor in and of itself for the onset of OP and that OP prevalence rises yearly with age. However, this study did not find a correlation between the risk of ABM and menopausal years, which may be due to the following factors: (1) OP activation of Wnt/β catenin signaling was accompanied by stress activation of the OPG/RANK/RANKL-related osteoblast pathway, which compensatively stimulated osteoblast formation; (2) Different SNPs on the same gene responded differently to environmental factors.

Several limitations of this study should be considered: (1) More studies are needed to confirm our results, especially in larger and different ethnic groups; (2) The study should include more SNPs of LRP5 and LRP6 genes; (3) The mechanisms affecting the reduction of BMD are complex, and more environmental factors affecting BMD should be included in the further study.

Research on the occurrence of ABM has received considerable attention in recent years, our research will increase understanding of the etiology of OP and provide a theoretical basis for the pathogenesis of OP.

## Conclusion

These findings indicate that LRP5-rs2306862 and LRP6-rs2302685 polymorphisms and gene–gene and gene-age interactions may increase the risk of ABM in postmenopausal women. There was no significant association between any of the SNPs and menopausal years and ABM susceptibility.

## Data Availability

All data generated or analyzed during this study are included in this published article.

## Abbreviations

LRP5: Low-density lipoprotein receptor-related protein 5
LRP6: Low-density lipoprotein receptor-related protein 6
ABM: Abnormal bone mass
SNPs: Single-nucleotide polymorphisms
BMD: Bone mineral density
OP: Osteoporosis
BTMs: Biochemical markers of bone turnover
BMI: Body mass index
WHR: Waist-to-hip ratio
ALP: Alkaline phosphatase
FN: Femoral neck
DEXA: Dual-energy X-ray
MDR: Multi-factor dimensionality reduction
